# Mechanical and Biological Characterization of Ionic and Photo-Crosslinking Effects on Gelatin-Based Hydrogel for Cartilage Tissue Engineering Applications

**DOI:** 10.3390/polym16192741

**Published:** 2024-09-27

**Authors:** Gabriele Boretti, Hafsteinn Esjar Baldursson, Luca Buonarrivo, Stina Simonsson, Sigurður Brynjólfsson, Paolo Gargiulo, Ólafur Eysteinn Sigurjónsson

**Affiliations:** 1 School of Science and Engineering, Reykjavik University, 102 Reykjavik, Iceland; esjarbaldurs@gmail.com (H.E.B.); luca.buonarrivo@gmail.com (L.B.); paolo@ru.is (P.G.); oes@ru.is (Ó.E.S.); 2 Institute of Biomedical and Neural Engineering, Reykjavik University, 102 Reykjavik, Iceland; 3 Institute of Biomedicine, Department of Clinical Chemistry and Transfusion Medicine, University of Gothenburg, 405 30 Gothenburg, Sweden; stina.simonsson@gu.se; 4 Faculty of Industrial Engineering, Mechanical Engineering and Computer Science, University of Iceland, 102 Reykjavik, Iceland; sb@hi.is; 5 The Blood Bank, Landspitali—The National University Hospital of Iceland, 101 Reykjavik, Iceland

**Keywords:** biopolymers, gelatin methacryloyl, hyaluronic acid methacrylate, cartilage tissue engineering, biomechanics, human adipose derived stem cells, scaffolds, regenerative medicine

## Abstract

Articular cartilage degeneration poses a significant public health challenge; techniques such as 3D bioprinting are being explored for its regeneration *in vitro.* Gelatin-based hydrogels represent one of the most promising biopolymers used in cartilage tissue engineering, especially for its collagen composition and tunable mechanical properties. However, there are no standard protocols that define process parameters such as the crosslinking method to apply. To this aim, a reproducible study was conducted for exploring the influence of different crosslinking methods on 3D bioprinted gelatin structures. This study assessed mechanical properties and cell viability in relation to various crosslinking techniques, revealing promising results particularly for dual (photo + ionic) crosslinking methods, which achieved high cell viability and tunable stiffness. These findings offer new insights into the effects of crosslinking methods on 3D bioprinted gelatin for cartilage applications. For example, ionic and photo-crosslinking methods provide softer materials, with photo-crosslinking supporting cell stretching and diffusion, while ionic crosslinking preserves a spherical stem cell morphology. On the other hand, dual crosslinking provides a stiffer, optimized solution for creating stable cartilage-like constructs. The results of this study offer a new perspective on the standardization of gelatin for cartilage bioprinting, bridging the gap between research and clinical applications.

## 1. Introduction

Articular cartilage is a specialized connective tissue that enables joint movement and distribution of loads, but it lacks the ability to self-repair due to the absence of blood vessels and nerves, leading to the progressive degeneration of defects and osteoarthritis [1,2]. Cartilage deterioration causes pain and functional impairments, often necessitating surgical intervention with inconsistent outcomes [3,4]. Given the limitations of current drugs and surgical materials, cartilage tissue engineering (CTE) offers a promising alternative [4,5]. An emerging strategy in CTE aims to develop scaffolds that replicate the properties of native cartilage tissue [6]. 3D bioprinting is a technology that extrudes living cells within printable bioinks, enabling the creation of customized tissue constructs [7,8,9]. This method is particularly effective due to its ability to produce patient-specific scaffolds with complex shapes and gradients of cells and biomaterials. The success of 3D bioprinting lies in the development of suitable bioinks and printing techniques for specific applications [10,11].

3D printed structures must meet key mechanical and biological requirements depending on the tissue they aim to replicate. The aggregate modulus of articular cartilage typically ranges from 0.1 to 3 MPa, where its compressive strength varies from 14 to 59 MPa and Poisson’s ratio from 0.06 to 0.30 [12,13,14,15,16]. Printed constructs must also combine structural stability with biological functionality to facilitate cell proliferation, new tissue formation, and controlled degradation [17,18]. Bioprinting materials are highly varied and need to be tailored to meet specific application requirements for optimal performance [19,20]. Hydrogels derived from natural biopolymers are extensively studied due to their biocompatibility, controlled cellular adhesion, and biodegradability [11,21]. Gelatin-based bioinks are particularly relevant in CTE due to the high collagen content in native cartilage [22,23]; however, they face challenges such as poor mechanical stability and rapid dissolution at physiological temperatures. Modifying gelatin molecules such as gelatin methacrylate (GelMA) to enable post-printing crosslinking addresses some of these issues [11]. GelMA bioinks offer low immunogenicity and high cell adhesion but suffer from brittleness and rapid degradation [4]. To improve GelMA’s performance in bioprinting, it is typically blended with natural biopolymers like alginate and xanthan gum, forming multicomponent hydrogels with enhanced viscosity and scaffold flexibility. This is also achieved by adjusting the concentration and crosslinking degree via divalent cation concentrations (e.g., Ca^2+^) [24,25,26,27].

Crosslinking is crucial in the formation of degrading hydrogel structures. In polymer chemistry, crosslinking stabilizes polymer chains by extending them into multidimensional networks, enhancing hydrogel stability and structure [28]. Literature reviews analyzing various crosslinking methods for gelatin-based biomaterials highlight the role that the choice of crosslinking technique plays in achieving optimal tissue regeneration [29,30,31,32]. Different methods can significantly influence the mechanical properties, biocompatibility, and structural integrity of the biomaterial, ultimately determining the success of the regenerated tissue. Therefore, selecting the appropriate crosslinking strategy is fundamental for tailoring the material’s properties to meet the specific requirements of the target tissue. Hydrogels are commonly crosslinked through photopolymerization or ionic methods. Photopolymerization is a rapid method for crosslinking hydrogels modified with a photoinitiator using a light source. Common photoinitiators like Irgacure 2959 and LAP generate free radicals under UV or visible light, forming crosslinked networks. Factors such as photoinitiator concentration, light intensity, and exposure time are key in controlling hydrogel properties, as they can also affect cell viability [11,33,34,35]. In contrast, ionic-crosslinking involves polymer interactions through ions and proteins rather than ionic bonds [36]. This process is simple and cost-effective but can lead to weaker mechanical properties and unpredictable degradation [37]. Dual-crosslinking, which combines light-induced and ionic methods, may improve scaffold stability and cell viability compared to single techniques [38,39].

The novelty of this study is the reproducible investigation of different crosslinking methods effects over a commercial gelatin-based bioink, aiming to optimize the printing process parameters to regenerate cartilage using 3D bioprinting. In detail, we investigated eight different crosslinking conditions to characterize the mechanical and biological properties of the printed construct for CTE applications. We assessed their mechanical properties using a compression assay and biological properties with a Live/Dead viability assay based on established protocols and literature.

## 2. Materials and Methods

To investigate the effects of different crosslinking methods on gelatin-based bioinks, we utilized the commercially available GelXA Cartilage bioink (Cellink, Gothenburg, Sweden). This hydrogel is primarily composed of GelMA, supplemented with sodium alginate, xanthan gum, hyaluronic acid methacrylate, and laminin 521, which is altogether specifically formulated to replicate the cartilage tissue microenvironment. The incorporation of hyaluronan and laminin 521 enhances the bioink’s cytocompatibility and promotes chondrogenic differentiation, while the methacrylated components (45–55% degree of methacrylation for GelMA and 15–25% for HAMA) and addition of LAP facilitate crosslinking through both photocuring and ionic methods.

### 2.1. Crosslinking Experiment Set Up

As illustrated in Table 1, the experimental setup included key evaluations of 3D-printed constructs under various crosslinking conditions. All tests were conducted in triplicate to ensure statistical reliability. A total of *n* = 73 samples were printed and tested: six for each mechanical condition, which was assessed on the day of printing and one week following incubation, and three for biological assessments, which were performed 24 h after printing.

The crosslinking conditions adopted in our experiments were based on the protocol provided by Cellink, on results from preliminary studies and on previous works in the literature that have employed similar bioinks, which guided our choice of exposure times [40,41,42,43]. This experimental variations allowed us to better understand the impact of different crosslinking durations on the final constructs. The chosen crosslinking conditions were as follows:

- **Ionic-crosslinking**: After printing, the constructs were kept in a 50 mM calcium chloride solution for either 5 or 15 min, depending on the experimental condition. They were then washed with PBS and placed in incubator with media.
- **Photo-crosslinking**: During printing, the constructs were exposed to 405 nm blue light for 15 s, either after each layer or every two layers, depending on the experimental condition being tested. The light source was positioned at a distance of 5 cm from the constructs.
- **Dual-crosslinking**: Constructs underwent a combination of both methods, with the blue light exposure during printing followed by submersion in the Crosslinking Agent. A total of four conditions were tested.

### 2.2. Bioprinting Process Parameters

The INKREDIBLE+ 3D bioprinter (Cellink, Sweden) was used to print cylindrical scaffolds, both with and without embedded cells. This printer features built-in photocuring modules (365 nm and 405 nm), enabling photo-crosslinking during or after bioprinting.

#### 2.2.1. G-Code Formulation

**Figure 1:**
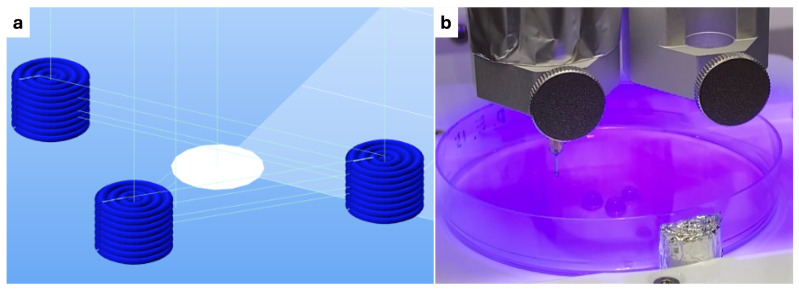
Triangular layout of three samples in a petri dish, crosslinked with centrally positioned light for equal exposure. (**a**) GCode interface from Repetier-Host Mac. (**b**) Photo-crosslinking of three samples in petri dish during the printing.

To create a structure that could replace the defect taken from a biopsy punch, a cylinder with a 4 mm diameter and 3 mm height was designed using Fusion360 CAD software (Autodesk, San Rafael, CA, USA). The STL file was sliced using HeartWare (Cellink, Sweden) using “Tissue Model” settings. The GCode was then adapted for the specific tests. For the photo-crosslinking experiment, three structures were arranged in a triangular pattern and photo-crosslinked with light positioned at the center, ensuring equal exposure for each sample, as shown in Figure 1.

#### 2.2.2. Printing Protocol

Preparation, printing, and sample handling were performed following Cellink’s protocol under sterile conditions in a safety cabinet. For the preparation of samples, the bioink was mixed with one-tenth of its volume of medium using two 3 mL syringes connected by a luer lock, either with or without cells, depending on the experiment. After thorough mixing, the bioink was incubated at 37 °C, transferred to a UV-shielded cartridge while carefully avoiding bubbles, then loaded into the print head set to 25 °C. Extrusion was carried out using a 23 G blunt needle (Cellink, Sweden). Preliminary tests were conducted to optimize the extrusion pressure and identify any potential issues. The final extrusion settings were: 80 kPa pressure and a temperature of 25 °C. Since the printer lacked temperature-insulated print heads, the cartridge was wrapped in aluminum foil to minimize heat loss, and the petri dish was cooled before starting each printing. Following printing, samples intended for ionic-crosslinking (or dual-crosslinking) were detached from the petri dish and transferred to a 24-well plate using sterile spatula and tweezers. The wells were then filled with cell culture medium and placed in an incubator.

### 2.3. Human Mesenchymal Stem Cell Culture

Human Adipose Derived Stem Cells (hADSCs) were used at passage #3. Cells were expanded in standard medium composed of DMEM-F12 with 10% pathogen inactivated platelet lysate (PIPL) supernatant and 1% Penicilin-Streptomycin. After two passages, hADSCs were trypsinized and counted in a hemocytometer using Trypan Blue staining to evaluate the number of dead cells. Cells and reagents were purchased from Thermo Fisher Scientific, MA, USA. 5 million of hADSCs were used for the experiment and resuspended in 100 μL to be then mixed with 1 mL of bioink. After printing, cell laden samples were kept in incubator in a 24-well plate. 2 mL of standard growing medium were changed in each well every other day.

### 2.4. Mechanical Characterization

Mechanical testing included unconfined compression tests conducted immediately after printing and one week later to assess the constructs’ stability and durability. The tests were performed using a Univert testing machine (CellScale, Waterloo, ON, Canada) equipped with a 20 N load cell and controlled by UniVert software. This setup is sensitive enough to measure mechanical properties of hydrogels intended for use in CTE [44,45,46,47]. The goal of these tests was to compare the stiffness of gelatin samples across different crosslinking conditions.

#### Compression Protocol and Mechanical Data Analysis

Three samples per crosslinking condition were kept in growth medium at 37 °C until testing and then analyzed. Each sample underwent three compressive loads at 50% deformation at a speed of 1 mm/min, aiming to evaluate the solid phase of the material while minimizing the liquid phase’s effect. The raw data (force and displacement) was cleaned and filtered to remove noise and inconsistencies. Stress-strain curves were generated based on sample dimensions and truncated at the highest stress point of the first cycle. The compressive modulus was calculated from the slope of the linear phase (20–80% of the max stress measured) of the stress-strain curves. The entire process, including data extrapolation and mechanical analysis, was conducted using MATLAB (MathWorks, Natick, MA, USA), which provided a robust platform for handling and processing the data, while statistical analysis was performed using RStudio.

### 2.5. Biological Characterization

Bioprinted samples were tested for biological assessment 24 h after-printing to measure the influence of the process parameters and different crosslinking methods.

#### 2.5.1. Live/Dead Assay and Images Analysis

The LIVE/DEAD™ Viability/Cytotoxicity Kit (ThermoFisher, Waltham, MA, USA) was used to assess cell viability, aligning with standard practices in the literature [48,49]. Samples were washed with PBS and incubated with the staining solution for 30 min. A negative control was prepared by incubating cells with methanol for 30 min. Samples were analyzed using a confocal microscope (OLYMPUS FV1200) with excitation at 488 nm and emission at 488 nm for green and 647 nm for red. Three acquisitions per sample were taken at three random locations in the 3D structure using a 10× magnification, and the imaging software utilized was cellSens, from Olympus. Confocal images were analyzed with Fiji’s particle analyzer plug-in [50]. The threshold for including particles was set to no larger than 5 microns, which facilitated counting the total number of cells (green + red cells) and the percentage of those alive (green cells) in each image. A Shapiro-Wilk test confirmed normal distribution of the data, and two-way ANOVA was used to identify significant differences between crosslinking conditions and samples from each condition, with a significance level set at *p* < 0.05.

## 3. Results and Discussion

Gelatin-based bioinks have shown promising results in CTE for their good biological and mechanical properties [51,52]. The addition of chondrogenic tissue specific components such as hyaluronic acid and laminin ensure a good environment for cartilage development; however, gelatin can be challenging to work with, and there is no established standard for its optimal crosslinking. For this reason, this work focused on exploring the effects of crosslinking on cell viability and mechanical properties of bioprinted constructs, with the aim to establish a standard for gelatin-based bioprinting.

### 3.1. Mechanical Properties and Construct Stability

**Figure 2:**
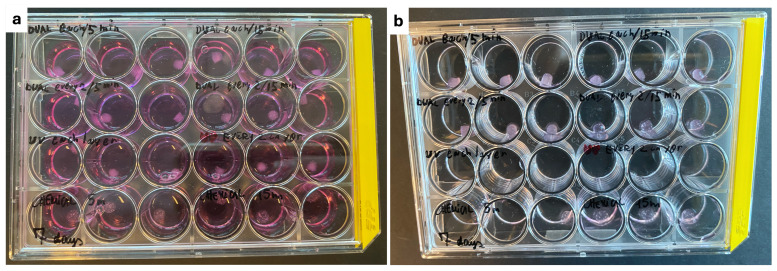
Printed samples in 24-well plate; (**a**) immediately after printing and (**b**) after four days of incubation, showing the dissolved state of the photo-crosslinked samples in the third row of the plate.

The first printing experiments indicated that the biomaterial was highly temperature-dependent, which was characterized by its small printability window; the bioink was too liquid to be printed at temperatures above 26 degrees and too solid to be extruded under 24 degrees. Moreover, samples differed in consistency depending on the length of time they were kept outside of the incubator, which highlights the influence of temperature on its viscoelastic properties. Notably, after four days (two medium changes), photo-crosslinked samples had completely dissolved, as shown in Figure 2. After one week of incubation, the remaining samples lost mechanical stiffness and could not be measured with the 20 N loading cell. Nevertheless, this bioink behaviour of degradability could potentially benefit CTE applications when cells are embedded in the matrix and expected to reproduce and substitute extracellular matrix (ECM). Indeed, researches support that controlled biodegradability can be a beneficial feature, aiding tissue growth while the scaffold progressively degrades [53,54].

Measures that would possibility overcome the dissolution of the samples could be the integration of cells into the scaffold, since for these mechanical tests we did not seed any cells into the scaffold, and we would expect cells to interact with the bioink and form extracellular matrix. If this would’t solve the problem, another possibility would be integrate the photo-crosslinking to a secondary crosslinking mechanism to enhance stability and mitigate early dissolution. In the end, only dual-crosslinking samples were sufficiently robust to be measured by our testing equipment.

#### Compressive Modulus after Printing

**Figure 3:**
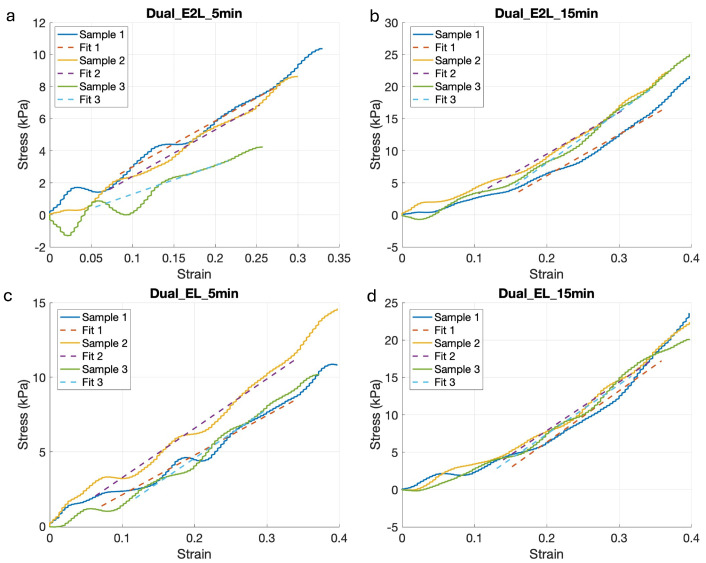
Mechanical results; stress-strain curves of the four measured crosslinking conditions and their correspective linear regressions for (**a**) photo-crosslinking every 2 layers with 5 min of chemical-crosslinking and (**b**) 15 min of chemical-crosslinking, (**c**) photo-crosslinking every layer with 5 min of chemical-crosslinking and (**d**) 15 min of chemical-crosslinking.

Results from the four measured conditions are presented in Figure 3, where compression tests confirmed the influence of the crosslinking method on the mechanical properties of the final structure. In particular, there was an evident difference in stress capacity between samples that were ionic-crosslinked for 5 min, compared to the samples that were ionic-crosslinked for 15 min, which were able to sustain higher stresses.

**Figure 4:**
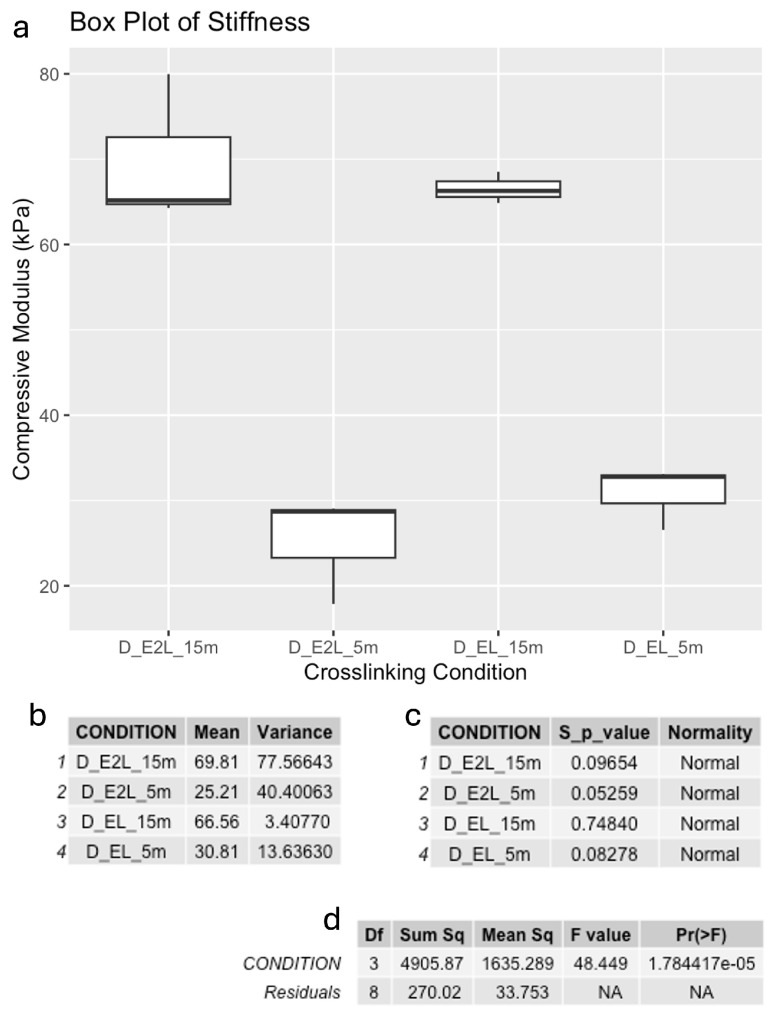
Mechanical results; including (**a**) box plot of the compressive moduli calculated for the four crosslinking conditions, (**b**) data summary, (**c**) Shapiro-Wilk test results for normality, and (**d**) ANOVA results for statistical significance of crosslinking methods.

The compressive modulus was calculated as the slope of the regression in the linear region of these curves (Figure 3). These results are presented as box plots in Figure 4. As illustrated, the compressive modulus was as high as 70 kPa for the stiffer condition, down to 25 kPa for the softer condition, which were photo-crosslinked every two layers and kept for 5 min in the calcium chloride solution. The increase in the compressive modulus with higher crosslinking is due to the denser network of polymer chains formed, which restricts molecular motion and increases the hydrogel stiffness. Longer exposure to the Crosslinking Agent facilitated stronger interactions between the polymer chains, resulting in higher compressive modulus. Moreover, we observed a positive correlation with the degree of crosslinking and compressive strength, as the scaffolds with longer crosslinking exposures resulted able to withstand higher compressive forces before breaking, as it is possible to see from the measured Stress in Figure 3. These compressive modulus values are consistent with other studies employing gelatin/hyaluronic acid-based hydrogels [55,56,57].

Normal distributions were validated with the Shapiro-Wink test, and one-way ANOVA determined whether differences in mechanical properties among crosslinking conditions were statistically significant (Figure 4).

### 3.2. Viability

A Live/Dead assay was performed 24 h after printing to evaluate the influence of the different crosslinking conditions on hADSC viability. Results showed high viability in all tested conditions.

**Figure 5:**
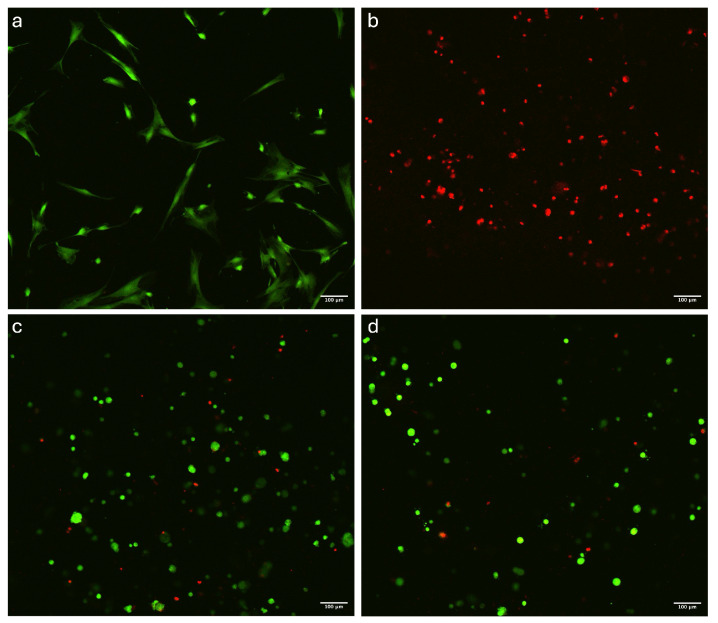

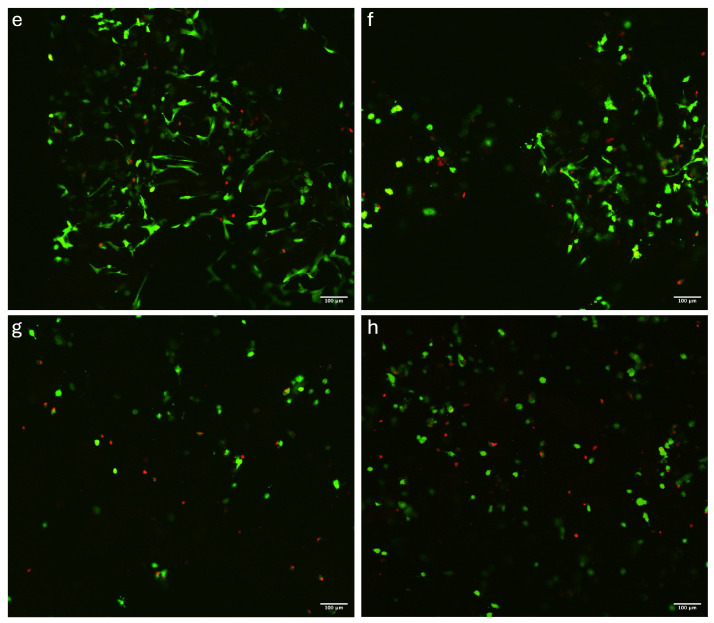
Confocal microscopy; Live/dead fluorescent assay showing (**a**) 2D positive control, (**b**) 3D negative control, (**c**) 5 min and (**d**) 15 min of exposure in ionic-crosslinking, (**e**) every two layers and (**f**) every layer in photo-crosslinking, (**g**) every two layers with 15 min of ionic-crosslinking, and (**h**) every layer with 15 min of dual-crosslinking. Image bar scale = 100 μm.

**Figure 6:**
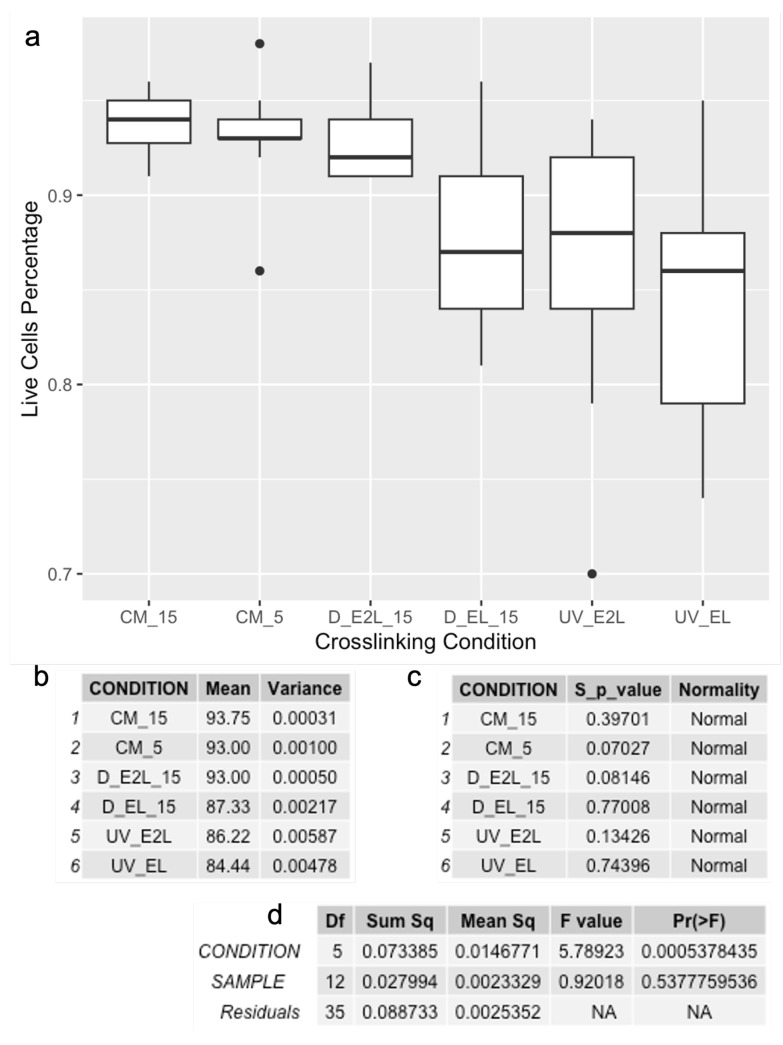
Viability results; including (**a**) box plot of cell viability across different conditions, (**b**) data summary, (**c**) Shapiro-Wilk test results for normality, and (**d**) Two-Way ANOVA results for statistical significance of crosslinking methods.

Overall, viable cells (stained green) were observed at high densities and were homogeneously distributed across all imaged areas (Figure 5). The cells notably displayed a predominantly spherical shape in the constructs crosslinked by ionic-crosslinking (Figure 5c,d) and dual-crosslinking (Figure 5g,h), while the photo-crosslinked samples (Figure 5g,h) had a stretched morphology, likely due to the higher inconsistency of the material. The successful colonization of the constructs by cells was further confirmed by cell density analysis, which was performed using Fiji software as described in Section 2.5.1. ANOVA results indicated statistically significant differences between the crosslinking conditions (*p* < 0.05), highlighting the impact of crosslinking methods on cellular viability (Figure 6). In contrast, differences between samples were not significant, indicating that the three samples for each condition were comparable. Results from cell counting indicated that all conditions supported high cell viability, with averages above 84%.

As expected, ionic-crosslinking yielded the highest viability of around 93%, while prolonged exposure to blue light resulted in a slight decrease in cell viability. The highest exposure yielded a viability of around 84%. The negative impact on cell viability observed in dual-crosslinking systems is possibly due to the increased crosslinking density, which can limit nutrient diffusion and increase local stiffness, making the environment less favorable for cell survival. To counter this, systems such as perfusion bioreactors could be used to improve the nutrient distribution [58].

## 4. Conclusions

The objective of this study was to evaluate the effects of various crosslinking techniques on gelatin-based bioinks. Measurements revealed that samples crosslinked using only photocuring lacked stability at 37 °C in growth medium, as they dissolved after two medium changes. In contrast, samples crosslinked solely with ionic methods retained their shape, but they were not stiff enough for mechanical testing and were challenging to 3D print due to layer flattening during the process. However, samples subjected to dual-crosslinking exhibited greater stability and improved mechanical properties, with reduced light exposure providing the most cell-friendly environment. Findings are summed up in Table 2.

The most promising crosslinking methods for CTE applications are the ones that combine photo-crosslinking (which allows for the manufacturing of 3D structures without having them collapsing during the printing process) with ionic-crosslinking, which improves their overall mechanical stability. These findings provide valuable insights into the optimal process parameters for printing gelatin-based materials like GelXA cartilage. This study further highlights the link between mechanical and biological properties of this bioink, paving the way for various applications in CTE. Additionally, when planning a study with this bioink, it is essential to consider its narrow printability window. A system capable of precisely controlling the print head temperature is important, along with maintaining a low printing bed temperature to ensure good shape fidelity. Further studies should continue to standardize mechanical characterization and viability methods, such as the ones performed in this study, to improve the comparability and reproducibility of these constructs. Further developments for improved cellular activity and mechanical stability might aim to include smart materials that are able to change their properties depending on external stimuli, or the use of dynamic culture conditions where bioreactors play an important role in improving nutrient distribution, waste removal, and mechanical stimulation [59,60,61].

## Figures and Tables

**Table 1:** Overview of experimental conditions, crosslinking methods, and tests.

Ionic		Photo		Dual			
Exposure	N. of Samples	Exposure	N. of Samples	Exposure	N. of Samples	Exposure	N. of Samples
5 min	6 for mechanical 3 for biological	15 s every layer	6 for mechanical 3 for biological	15 s every layer + 5 min ionic	6 for mechanical 3 for biological	15 s every 2 layers + 5 min ionic	6 for mechanical 3 for biological
15 min	6 for mechanical 3 for biological	15 s every 2 layer	6 for mechanical 3 for biological	15 s every layer + 15 min ionic	6 for mechanical 3 for biological	15 s every 2 layers + 15 min ionic	6 for mechanical 3 for biological

**Table 2:** Summary of results for each experimental condition, highlighting the compressive modulus, cell viability, and overall considerations observed across different crosslinking methods.

Crosslinking	Compressive Modulus	Cellular Viability	Overall
Ionic 5 m	Not measurable	~93%	Enhanced cellular compatibility, with reduced structural stability during the printing process
Ionic 15 m	Not measurable	~94%	Enhanced cellular compatibility, with reduced structural stability during the printing process
Photo every layer	Not measurable	~84%	Structural stability during printing, with complete dissolution observed after 4 days and lower viability
Photo every 2 layer	Not measurable	~86%	Structural stability during printing, with complete dissolution observed after 4 days and lower viability
Photo every layer + ionic 5 m	~20 kPa	Not measured	Extended preparation time, with favorable mechanical properties
Photo every layer + ionic 15 m	~43 kPa	~87%	Extended preparation time, with good mechanical properties
Photo every 2 layer + ionic 5 m	~16 kPa	Not measured	Extended preparation time, with favorable mechanical properties
Photo every 2 layer + ionic 15 m	~45 kPa	~93%	Extended preparation time, with enhanced mechanical properties and higher cell viability

## Data Availability

Data is contained within the article.
